# Variation in early‐life maltreatment effects on adolescent psychopathology due to contamination

**DOI:** 10.1002/jts.70076

**Published:** 2026-05-08

**Authors:** Emily D. Dunning, Chad E. Shenk, Zachary Fisher, Steffany J. Fredman, John Felt

**Affiliations:** ^1^ Department of Human Development and Family Studies The Pennsylvania State University University Park Pennsylvania USA; ^2^ Department of Psychology University of Rochester Rochester New York USA; ^3^ Mt. Hope Family Center University of Rochester Rochester New York USA; ^4^ Department of Quantitative Psychology University of North Carolina at Chapel Hill Chapel Hill North Carolina USA; ^5^ Center for Healthy Aging The Pennsylvania State University University Park Pennsylvania USA

## Abstract

This study examined the effect of early‐life maltreatment on adolescent psychopathology and whether the significance and magnitude of this effect varied based on the presence of contamination (i.e., when individuals in a comparison group are exposed to maltreatment). Data were drawn from the Longitudinal Studies of Child Abuse and Neglect (LONGSCAN; *N* = 1,354), a prospective cohort of children at risk for maltreatment. Children in the study were recruited by the time they were 4 years old (*M*_age_ = 4.56 years, *SD* = 0.70), and the sample was 50.8% female and 51.8% Black (25.7% White, 8.0% Hispanic, 1.0% Native American, 0.5% Asian, 11.3% mixed race, 1.7% other). Confirmed indicators characterized maltreatment prior to and after age 4 years. Adolescent self‐report indicators identified maltreatment prior to 16 years of age and corrected contamination. Psychopathology was assessed via self‐report at the LONGSCAN Age 16 assessment. Propensity score analyses indicated that maltreatment prior to 4 years of age only predicted significantly greater adolescent psychopathology symptoms after correcting contamination, *B =* 6.40, 95% CI [2.02, 10.79], *SMD* = 0.32, *p* = .004. Further, effect magnitudes increased 67% by correcting contamination. The results were robust to adjustments for maltreatment reexposure and covariate quality informing propensity score analyses. Correcting contamination can aid the discovery and replication of the effects of early life maltreatment on psychopathology.

Decades of research have demonstrated that child maltreatment (CM), when examined globally and by each major subtype, is robustly associated with an increased risk of multiple forms of psychopathology, including mood, anxiety, trauma‐related, and conduct disorders (Jaffee, 2017; Mills et al., 2013). These disorders are highly comorbid in the CM population (Widom et al., 2007), with symptom severities often exceeding those in the general population (Teicher & Samson, 2013). Moreover, these heterogeneous and severe presentations can result in symptoms that persist from childhood well into adulthood (McLaughlin et al., 2010). Establishing a strong evidence base for the risk for psychopathology following CM exposure is, therefore, critical for informing policies on the need for prevention and treatment services as well as the optimal time in development at which to deliver those services.

Although existing research supports the direction of effects for CM on psychopathology, there remains considerable variation in the statistical significance and magnitude of these effects across studies (Hankin, 2005; Keyes et al., 2012). This is particularly true for research examining the effect of early‐life maltreatment on psychopathology in adolescence. Exposure to CM most often occurs between birth and 4 years of age (U.S. Department of Health and Human Services, 2024), a developmental period that may have a greater impact on the onset, severity, and persistence of psychopathology given the environmental sensitivity of biobehavioral regulatory processes during the first few years of life (Juster et al., 2011). Studies have found that individuals with an earlier onset of CM (i.e., 0–5 years old) have more adjustment problems (Keiley et al., 2001) and internalizing and externalizing behaviors in adolescence (Duprey et al., 2017), with more severe anxiety and depressive symptoms in young adulthood, relative to those with later maltreatment onset (Kaplow & Widom, 2007). However, other studies have shown that children maltreated in middle childhood (ages 6–10 years) or adolescence (age 13 years and older) evidence a significantly higher risk of emotion dysregulation and depressive symptoms relative to those who were maltreated in childhood (Capretto, 2020; Dunn et al., 2018). In fact, some studies indicate that individuals exposed to early‐life maltreatment do not have a higher risk of internalizing or externalizing symptoms in adolescence relative to their nonmaltreated peers (Thornberry et al., 2001). Thus, variation in the statistical significance and magnitude of early‐life maltreatment effects makes conclusions about whether such maltreatment uniquely impacts later psychopathology and, therefore, warrants special consideration as a developmental window for intervention, tentative.

One factor that may explain variation in the statistical significance and magnitude of effect for early‐life maltreatment on adolescent psychopathology is the presence of contamination. Contamination is a methodological phenomenon that occurs in both randomized and observational research wherein members of a control or comparison group use, receive, or are exposed to a treatment under investigation (Cuzick et al., 1997; Delgado‐Rodríguez & Llorca, 2004). The presence of contamination poses a threat to the estimation of treatment effects because observed levels of an outcome for individuals in a control group will more closely approximate levels of the same outcome for those in a treatment group. This results in a bias that minimizes the magnitude of between‐group differences and limits the detection of such differences at set levels of statistical significance (Keogh‐Brown et al., 2007). As the prevalence of contamination varies across studies, so will the extent of bias it has on treatment effects, leading to variation in the statistical significance and magnitude of those effects on outcomes.

In CM research, contamination occurs when individuals recruited to a non‐CM comparison group have been exposed to maltreatment (Shenk et al., 2023). Several studies have demonstrated the presence of contamination in CM research and how the resulting bias can lead to variation in the significance and magnitude of the effects of maltreatment. For example, Scott et al. (2010) examined the risk of psychiatric disorders in young adulthood in a nationally representative sample of individuals from New Zealand who were and were not the subject of a child protective services (CPS) investigation in childhood (*N* = 2,144). Contamination was identified as any case of self‐reported maltreatment for participants who were never the subject of a CPS investigation in childhood. When contamination was corrected by omitting these individuals from the statistical model, the magnitude of the effect for CM on past‐year and lifetime history of psychiatric disorders increased by 22%–32%, and the effect for maltreatment on several individual disorders became statistically significant. In a separate, multiwave study (*N* = 514), Shenk et al. (2016) examined the risks for a range of health outcomes (i.e., clinical levels of major depressive disorder, teenage births, obesity, and past‐month cigarette use) among female adolescents following exposure to substantiated CM. Statistical models that ignored contamination failed to obtain significant results for two of four outcomes (depression and obesity). However, correcting contamination by removing these individuals from the statistical model resulted in each outcome achieving statistical significance, with effect magnitudes increasing 24%–130%. Recent studies that correct contamination by removing individuals from the model (Felt et al., 2024) or by retaining all individuals and modeling contamination as a third group (Shenk et al., 2022) showed similar changes in the statistical significance and magnitude of the effects of CM. Thus, the presence of contamination, without correction, may be an important contributor to variation in the statistical significance and magnitude of the effects of early‐life maltreatment on adolescent psychopathology. Addressing contamination may lead to more consistent and replicable findings regarding the impact of early‐life CM on adolescent psychopathology.

CM is also a time‐varying phenomenon that often occurs more than once throughout child development (Russotti et al., 2021). Thus, it is possible that reexposure to maltreatment after early‐life exposure contributes to the associations between early‐life maltreatment and adolescent psychopathology symptoms, making it impossible to know whether the effects of maltreatment on psychopathology are due to exposure in early life, later life, or both. Research has demonstrated a significant impact of reexposure on the links between CM and psychopathology while simultaneously considering developmental timing (English, Upadhyaya, et al., 2005). However, not all research on developmental timing has included reexposure. As such, variation in findings regarding developmental timing and the potential impact of reexposure limits conclusions about whether early‐life maltreatment has lasting effects on adolescent psychopathology, as well as the optimal developmental timing for delivering preventive and treatment services.

The current preregistered study examined contamination‐related variation in the effects of early‐life maltreatment on adolescent psychopathology, contributing to a relatively sparse literature on contamination in CM research. We examined psychopathology in adolescence as a test of the long‐term impacts of early‐life exposure to maltreatment, consistent with prior research (Duprey et al., 2017), because adolescence represents an important time to access intervention services prior to adulthood. We adopted the use of propensity score analysis to approximate the causal effect of early‐life maltreatment on adolescent psychopathology before and after correcting contamination. Robustness tests were conducted to determine whether adjustments for reexposure to CM after early life (Russotti et al., 2021) and the quality of covariates included in the propensity score analyses (Holmberg & Andersen, 2022) further altered the statistical significance and magnitude of CM effects.

## METHOD

### Participants and procedure

Data were drawn from the Longitudinal Studies of Child Abuse and Neglect (LONGSCAN; Runyan et al., 2014). LONGSCAN is a multiwave prospective cohort study (*N* = 1,354) examining the antecedents and consequences of CM in a diverse sample of children who experienced or were at risk for maltreatment. There were five data collection sites in varying regions of the United States—the Southwest, Northwest, Midwest, South, and East—that used common assessment protocols and can be leveraged as one study. Sites varied in recruitment criteria such that some sites recruited higher‐risk samples compared to others: The East site recruited children and their families from a pediatric clinic who were at high risk for CM based on demographic risk factors, the South and Midwest sites recruited children who had a CPS report for CM or were at high risk for CM, and the Northwest and Southwest sites recruited only children with a CPS report. Participants were recruited when the child was approximately 4 years old (*M*_age_ = 4.56 years, *SD* = 0.70), with families assessed biennially until the child reached 18 years of age. Herein, we refer to LONGSCAN assessments as Age 4, Age 6, and so on, through Age 18.

### Measures

#### Confirmed maltreatment

Independent raters at each LONGSCAN site used the Modified Maltreatment Classification System (MMCS; English, Bangdiwala, et al., 2005) to review official case records from CPS agencies. Case records, which were generated when there was an official investigation into an allegation of CM, were reviewed for each family at least every 2 years throughout LONGSCAN. Raters used the information from the case records to assess whether the information met a preset standardized definition of CM across LONGSCAN sites. The current study used MMCS ratings to create two indicator variables. The first variable represented any confirmed case of maltreatment exposure from ages 0–4 years (1 = yes, 0 = no), which established the maltreatment and comparison groups used in subsequent analyses. The second variable represented any confirmed case of maltreatment from ages 4–16 years (1 = yes, 0 = no), which was used to inform estimates of contamination and confirmed maltreatment reexposure. The MMCS has previously shown high convergent (κs = .72–.98; Runyan et al., 2005) and criterion validity with other measures of neglect and, in the present study, MMCS raters met a 90% interrater reliability threshold for reviewing CPS case records.

#### Self‐reported maltreatment

The Self‐Report of Physical, Sexual, and Psychological Abuse (SPSPA; Knight et al., 2000) was used to assess self‐reports of physical abuse, sexual abuse, and psychological abuse in the LONGSCAN sample. The SPSPA relies on the definitions of abuse developed by Barnett et al. (1993), and the development of SPSPA items was guided by MMCS‐defined caregiver behaviors and injury outcomes that would generally reach the thresholds used by CPS agencies in the United States. Adolescents completed the SPSPA at Age 12 to measure prior lifetime exposure to child abuse and again at Age 16 to assess abuse exposure since Age 12. An indicator variable was used to classify participants as self‐reporting at least one lifetime instance of child abuse before Age 16 (1 = yes, 0 = no). An examination of the psychometrics of the SPSPA in the LONGSCAN sample suggests good concurrent and predictive validity of the SPSPA in relation to child outcomes (Everson et al., 2008).

#### Self‐reported neglect

The About My Parents (AMP; LONGSCAN Investigators, 1998a) and Mid‐Adolescent Neglect Scale (ANM; LONGSCAN Investigators, 1998b) were completed to capture self‐reported instances of child neglect across four domains: physical needs, emotional support, monitoring/supervision, and educational support. The AMP is composed of 25 items, which were phrased positively (e.g., “How often did your parent(s) make sure that you saw a doctor when you needed one?”), and responses were rated on a 4‐point scale ranging from 0 (*never*) to 3 (*a lot*). The current study used AMP self‐reports of neglect measured at Age 12, which assessed neglect experienced in elementary school and in the last year, and at Age 14, which assessed past‐year neglect. Consistent with prior LONGSCAN research (Dubowitz et al., 2011), six items were dropped from the AMP measure due to issues with cross‐loading and poor model fit when including these items, resulting in a total of 19 items. All AMP items were then reverse‐scored such that higher ratings indicated a higher risk of neglect. Consistent with prior research (Springer et al., 2007), participants who endorsed the most severe rating on any AMP item were classified as having self‐reported neglect (1 = yes, 0 = no). For the current study, we then matched the 19 AMP items to the corresponding set of items on a separate self‐report measure of neglect, the ANM, that was administered at Age 16 to capture the occurrence of past‐year neglect. Using the same items ensured consistent measurement of self‐reported neglect at Ages 12, 14, and 16. The ANM item content and response scale matched those for the AMP, and participants who endorsed the most severe rating on ANM items were classified as having self‐reported neglect (1 = yes, 0 = no). Previous work on psychometric properties of the AMP and ANM suggests modest to moderate convergent validity with other measures of neglect (Dubowitz et al., 2011).

#### Contamination and maltreatment reexposure

An indicator variable was created to represent the presence of contamination, defined as any confirmed case of maltreatment occurring after age 4 or any self‐report of maltreatment at any time for those in the comparison group (yes = 1, no = 0). Maltreatment reexposure was defined as exposure to confirmed maltreatment between ages 4‐16 (yes = 1, no = 0) for those children who were exposed to maltreatment between ages 0‐4.

#### Trauma‐related psychopathology symptoms

The Trauma Symptom Checklist for Children (TSCC; Briere, 1996) is a 54‐item self‐report measure that is used to assess transdiagnostic psychopathology symptoms (e.g., how often the child has bad dreams or nightmares, how often the child feels afraid that something bad might happen to them). Responses are rated on a 4‐point scale ranging from 0 (*never*) to 3 (*almost all of the time*) and summed, with higher scores indicating higher levels of psychopathology symptoms. The TSCC total score has demonstrated good internal consistency in previous research (Cronbach's αs = .84–.97; Briere, 1996; Nilsson et al., 2008). The current study used TSCC total scores obtained at Age 16 as a transdiagnostic measure of psychopathology in adolescence (Morelli et al., 2021). In the present study, Cronbach's alpha was .96.

### Data analysis

Complete data on exposure to confirmed CM between the 0–16 years of age were obtained for the entire LONGSCAN sample. Participants with missing data on self‐reported maltreatment were excluded from the analyses. After excluding these participants, missing data for the remaining study variables were multiply imputed before conducting analyses. We used predictive mean matching with multiple imputation via the *mice* package in R version 2026.01.2 (R Core Team, 2023; van Buuren & Groothius‐Oudshoorn, 2011) across 25 datasets with 100 iterations each. Imputation quality was determined using diagnostic plots. Additionally, we used entropy balancing to adjust for stringent and poorer‐quality covariates in all models. Entropy balancing is a type of propensity score weighting that guarantees covariate mean balance and aims to minimize bias in the estimation of treatment effects (Hainmueller, 2012). Covariates were included in the entropy balancing step for each respective model. The quality of propensity scores was assessed using Kolmogorov–Smirnov (*KS*) statistics (standardized mean difference [*SMD*] < 0.1, *KS* < 0.1; Markoulidakis et al., 2023). For all models, results were pooled across all 25 datasets per Rubin's rules via the MatchThem package in R, which performs matching and weighting across these multiply imputed datasets (Pishgar et al., 2021). The average treatment effect for the treated (ATT) was targeted as the causal estimand (Austin, 2011), which, in the case of the present study, represents the difference between the average outcomes observed for children exposed to maltreatment and the average outcomes these children would have experienced had they not been maltreated. ATTs were estimated using multiple regression with the *lm* function in R after achieving balance on covariates and adjusting estimates for differences in psychopathology symptom severity across LONGSCAN sites. Alpha was set at the conventional .05 level, and effect sizes were evaluated based on SMDs between CM and comparison groups and interpreted according to Cohen's (1988) standards.

#### Covariates

Maltreatment and comparison groups were balanced on a stringent list of covariates in all models to control for various confounders. These covariates were selected because they either occurred before CM onset or were hypothesized not to be impacted by maltreatment, which is important covariate selection criteria when using propensity score methods (Kainz et al., 2017). The selected covariates were child age (in years) at the Age 4 LONGSCAN visit, child gender, child racial and/or ethnic minority status, caregiver age (in years) at the Age 4 LONGSCAN visit, yearly family income, and whether the caregiver reported experiencing physical or sexual abuse in childhood.

An expanded set of lower‐quality covariates was used in a robustness test to examine whether the effects of early‐life maltreatment on adolescent psychopathology differed when variables were included in the model that had unknown temporal associations with maltreatment exposure or when the influence of maltreatment exposure on a variable was unclear, features that can lead to potentially biased results (Holmberg & Andersen, 2022). The expanded list of covariates for the robustness test included all stringent covariates listed previously, as well as: the presence of a male partner in the home; family use of economic services; caregiver educational attainment; total weeks the child was separated from the caregiver; service use for child behavioral, emotional, or school problems; child health problems; child low birth weight; child preschool depressive symptoms; child exposure to violence in school; caregiver depressive symptoms; caregiver perceived social support; caregiver risk for alcoholism; caregiver physical health; and caregiver negative past‐year life events. Additionally, LONGSCAN site was included as a main effect in the final models due to significant differences in Age 16 psychopathology symptoms across data collection sites, *F*(4, 776) = 4.02, *p* = .003.

#### Primary models

We used a two‐step modeling approach to examine possible variations in the statistical significance and magnitude of the effect of early‐life CM on adolescent psychopathology due to contamination. Model 1 tested this association without correcting contamination, using a two‐level variable to predict levels of psychopathology. In Model 1, these groups were: (a) a maltreatment group, composed of participants exposed to confirmed maltreatment between 0 and 4 years of age, including those who had confirmed maltreatment between ages 4 and 16 years, and (b) an original comparison group composed of participants who were not exposed to confirmed maltreatment between ages 0–4 years but who may have been exposed to confirmed maltreatment between ages 4–16 years or self‐reported maltreatment at any point in their lifetime. Model 2 identified contamination and corrected it by removing individuals from the original comparison group and classifying them into a third group. This resulted in a three‐level variable used to predict levels of adolescent psychopathology: (a) an early‐life maltreatment group, composed of participants exposed to confirmed maltreatment between ages 0–4 years, including those who had confirmed maltreatment between ages 4–16 years; (b) a revised comparison group, in which participants had no confirmed or self‐reported CM between ages 0–16 years; and (c) a contamination group with participants who did not have a confirmed case of maltreatment between ages 0–4 years but were exposed to confirmed maltreatment between ages 4–16 years and/or self‐reported maltreatment at any age. See Table 1 for demographic and study‐related information across these three groups. The results from Model 2, therefore, provided an opportunity to evaluate change in the statistical significance and magnitude of effects for early‐life CM on adolescent psychopathology when contamination has been corrected.

**TABLE 1:** Sample demographic characteristics for maltreatment, contamination, and revised comparison groups

	Maltreatment			Contamination			Revised comparison		
	(*n* = 460)			(*n* = 448)			(*n* = 183)		
Variable	*M*	*SD*	%	*M*	*SD*	%	*M*	*SD*	%
Child age (years) at Age 4	4.40	0.53		4.67	0.77		4.57	0.74	
CG age at Age 4	37.24	11.21		30.77	9.07		29.05	7.28	
TSCC total score at Age 16	25.46	22.24		24.32	19.50		14.86	13.70	
Child gender(female)			54.8			47.8			52.9
Child racial/ethnic minority									
Racial and/or ethnic minority			71.4			76.3			77.8
Non‐Hispanic, White			28.6			23.7			22.2
CG exposed to CPA or CSA			68.2			43.3			40.0

*Note*: The median family income for all groups was $10,000–$14,000 (USD) at the Longitudinal Studies of Child Abuse and Neglect (LONGSCAN) Age 4 Assessment (Age 4). Age 16 = LONGSCAN Age 16 Assessment; CG = caregiver; TSCC = Trauma Symptom Checklist for Children; CPA = child physical abuse; CSA = child sexual abuse.

#### Robustness tests

We conducted two robustness tests to further interrogate the results obtained in Model 2. Model 3 estimated the effect of early‐life maltreatment on adolescent psychopathology after correcting both contamination and reexposure to maltreatment. Reexposure was corrected by excluding children who were first exposed to confirmed maltreatment between ages 0–4 years and then again during ages 4–16 years from the analysis. Model 4 repeated Model 2 but used the expanded set of covariates listed previously to assess whether the quality of covariate selection altered the results. See Figure 1 for a flow chart with corresponding sample sizes for primary models and robustness assessments. In sum, the results from Model 1 estimated the effect of early‐life CM on adolescent psychopathology without correcting contamination, Model 2 reestimated this effect after correcting contamination, Model 3 assessed the impact of maltreatment reexposure on estimates generated from Model 2, and Model 4 examined the impact of covariate quality on estimates generated from Model 2.

**FIGURE 1:**
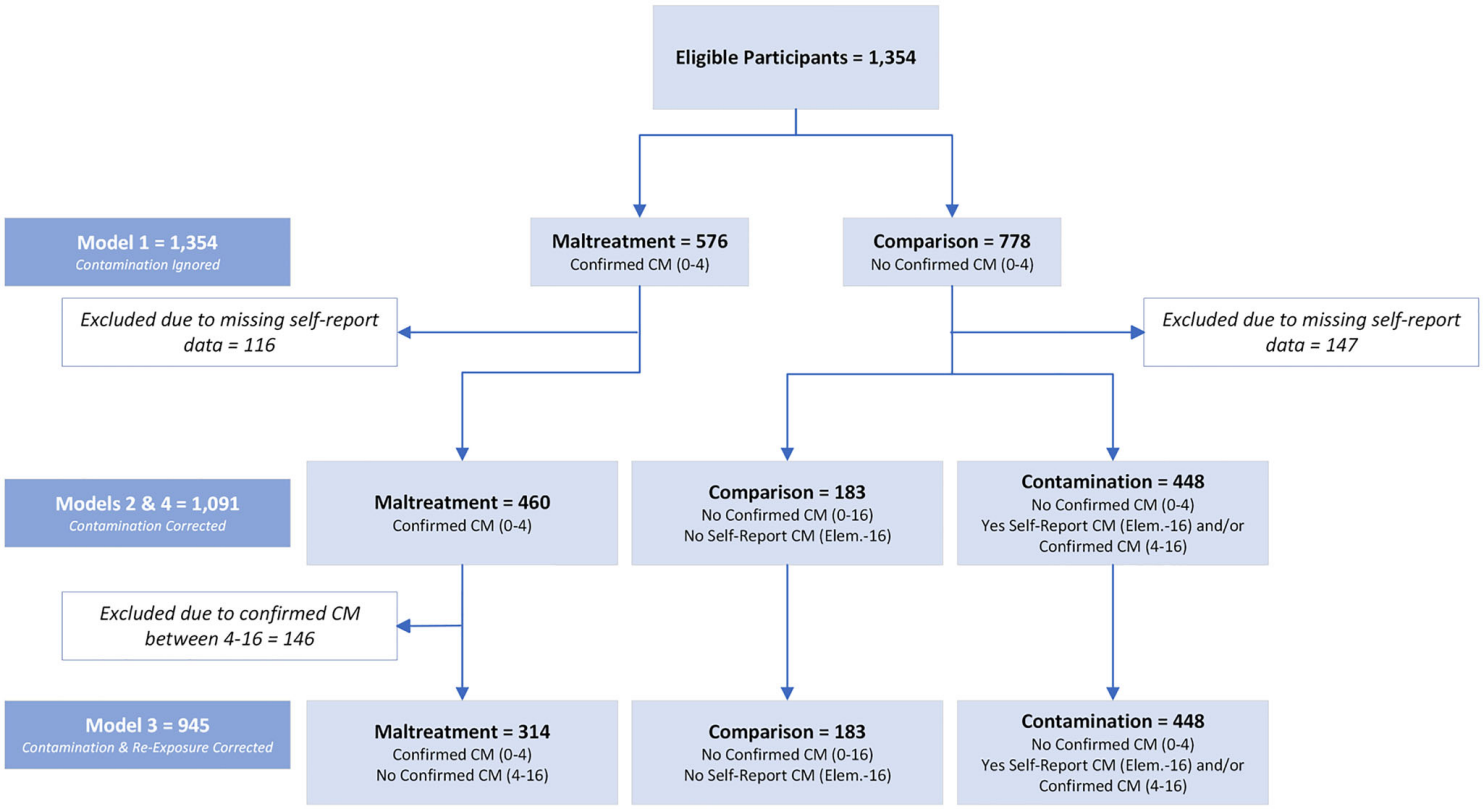
CONSORT diagram of group sizes moving from Models 1–4 *Note*: Moving from Model 1 to Models 2 and 4, participants were sorted into the contamination group because they self‐reported child maltreatment (CM; *n* = 341), had confirmed maltreatment between ages 4–16 years (*n* = 31), or had both a self‐report and confirmed maltreatment between ages 4–16 years (*n* = 76).

## RESULTS

### Prevalence of contamination

For participants with no confirmed case of CM in the first four years of life (*n* = 631), 448 self‐reported at least one instance of lifetime CM and/or had a confirmed case of maltreatment after Age 4. This resulted in a contamination prevalence of 71.0%.

### Primary models

#### Model 1: Contamination and reexposure ignored

After adjusting for site differences, there was no statistically significant effect of early‐life maltreatment on the severity of adolescent psychopathology in the regression model using a two‐level predictor (early life CM vs. original comparison), *B* = 2.11, 95% confidence interval (CI) [‐1.24, 5.47], *SMD* = 0.10, *p* = .215. There was a significant effect of site on psychopathology symptoms such that individuals from the Midwest, *B* = 5.14, 95% CI [0.68, 9.60], *SMD* = 0.25 *p* = .024; South, *B* = 4.89, 95% CI [0.34, 9.44], *SMD =* 0.24, *p = *.035; and Southwest, *B* = 5.60, 95% CI [0.44, 10.76], *SMD =* 0.28, *p = *.034, sites reported significantly higher levels of psychopathology symptoms than those from the East site.

#### Model 2: Contamination corrected, reexposure ignored

The regression model that corrected contamination by using a three‐level predictor (early life maltreatment, revised comparison, contamination) indicated a statistically significant effect of early‐life CM on the severity of adolescent psychopathology, *B =* 6.40, 95% CI [2.02, 10.79], *p = *.004, after adjusting for site differences, such that children exposed to maltreatment between 0–4 years of age scored, on average, 6.40 points higher on the TSCC than those in the revised comparison group. The magnitude of this effect was in the small range, *SMD =* 0.32, but it was statistically significant and 66.98% larger in magnitude relative to results from Model 1. Means and standard deviations for psychopathology symptoms are presented in Table 1, and pairwise contrasts for the three‐level predictor in Model 2 are reported in Table 2. There was a significant effect of site on psychopathology symptoms such that individuals from the Midwest, *B* = 5.41, 95% CI [0.97, 9.86], *SMD =* 0.27, *p = *.017, and South, *B* = 4.63, 95% CI [0.08, 9.17], *SMD =* 0.23, *p = *.046, sites had significantly higher levels of psychopathology symptoms than those from the East site.

**TABLE 2:** Propensity score analysis estimating psychopathology symptoms across all models

Variable	*B*	*SE*	*SMD*	95% CI	*p*
Model 1: Contamination uncorrected					
Maltreatment vs. original comparison	2.11	1.70	0.104	[‐1.24, 5.47]	.215
Model 2: Contamination corrected					
Maltreatment vs. revised comparison	6.40	2.23	0.315	[2.02, 10.79]	.004
Contamination vs. revised comparison	5.75	2.09	0.283	[1.64, 9.86]	.006
Maltreatment vs. contamination	0.66	1.56	0.032	[‐4.42, 1.72]	.388
Model 3: Robustness to maltreatment reexposure					
Maltreatment vs. revised comparison	5.53	2.25	0.285	[1.11, 9.94]	.014
Contamination vs. revised comparison	6.21	1.83	0.321	[2.61, 9.81]	< .001
Maltreatment vs. contamination	−0.68	1.81	−0.035	[‐4.25, 2.88]	.707
Model 4: Robustness to expanded covariates					
Maltreatment vs. revised comparison	5.74	2.41	0.279	[0.98, 10.49]	.018
Contamination vs. revised comparison	5.11	2.12	0.248	[0.92, 9.29]	.017
Maltreatment vs. contamination	0.63	1.74	0.030	[‐2.80, 4.06]	.717

*Note*: SMD = standardized mean difference; CI = confidence interval.

### Robustness checks

#### Model 3: Contamination and reexposure corrected

After contamination was corrected, there remained 146 participants in the early‐life maltreatment group who had experienced at least one subsequent confirmed case of maltreatment between the ages of 4 and 16 years. These individuals were removed from Model 3 to assess the effect of early‐life maltreatment on adolescent psychopathology after accounting for maltreatment reexposure. Model 3 results, using the three‐level predictor that corrected contamination in Model 2, appeared robust to adjustment for reexposure such that a statistically significant effect of early‐life CM on adolescent psychopathology remained, *B =* 5.53, 95% CI [1.11, 9.94], *p = *.014, resulting in an average of 5.53 more points on the TSCC for participants in the early‐life maltreatment group relative to the revised comparison group. The magnitude of this effect was in the small range, *SMD =* 0.29, and similar to results obtained in Model 2. Pairwise contrasts for the three‐level predictor in Model 3 are reported in Table 2. There were no significant effects of site on psychopathology symptoms in Model 3.

#### Model 4: Contamination corrected, reexposure ignored, and quality of covariates

Model 4 reestimated Model 2 using the three‐level predictor with the expanded list of covariates to assess the robustness of the results obtained when contamination was corrected and the criteria for the quality of covariates used in propensity score analysis were relaxed. The results were robust to the quality of covariates informing the propensity score analysis, such that after adjusting for site differences, there was a statistically significant effect of early‐life maltreatment on the level of psychopathology symptoms in adolescence once balance was achieved on the expanded set of covariates, *B* = 5.74, 95% CI [0.98, 10.49], *p = *.018. The results indicated that participants in the early‐life maltreatment group scored an average of 5.74 points higher on the TSCC than those in the revised comparison group. The magnitude of this effect was in the small range, *SMD =* 0.28, and similar to the estimate obtained in Model 2 with the restricted set of covariates. Complete pairwise contrasts for the three‐level predictor in Model 4 are reported in Table 2. There was also a significant effect of site on psychopathology symptoms such that individuals from the Midwest, *B* = 4.71, 95% CI [0.36, 9.06], *SMD =* 0.229, *p = *.034, and Southwest, *B* = 5.03, 95% CI [0.35, 9.71], *SMD =* 0.244, *p = *.035, sites had significantly higher levels of psychopathology symptoms than those from the East site.

## DISCUSSION

This study is the first of which we are aware to examine the role contamination plays in generating variation in the statistical significance and magnitude of the effect for early‐life maltreatment on adolescent psychopathology. This study adds to a growing body of research demonstrating that contamination exists in independent cohorts examining the health consequences of CM and that failure to correct contamination can make it more difficult to detect the effects of maltreatment and accurately estimate the strength of those effects. The present study reported a contamination prevalence of 71.0%, providing evidence that a majority of the original comparison group had experienced CM prior to Age 16. The prevalence of contamination in the present study appears to be an upper‐bound estimate compared to prior research (15%–67%; Felt et al., 2024; Scott et al., 2010), which is likely due to the higher risk of maltreatment among individuals in the LONGSCAN cohort. Ignoring or failing to correct for such contamination can have an impact on the estimation of the effects of CM. Indeed, the current study found a statistically significant effect of early‐life maltreatment on adolescent psychopathology only when contamination was corrected, contrary to previous literature (Jaffee, 2017; Mills et al., 2013). Once contamination was corrected, models identified a statistically significant effect for early‐life maltreatment on adolescent psychopathology that increased in effect size magnitude by 67%. This is in line with previous studies showing increases in effect magnitudes ranging from 17% to 130% after correcting contamination (Felt et al., 2024; Shenk et al., 2016). The results of this study support prior research suggesting contamination exists in observational research on CM and that failure to correct contamination can result in the inability to detect a statistically significant effect for maltreatment and, even when an effect is detected, an underestimation of the magnitude of CM effects on psychopathology.

We also examined whether these primary results were robust to adjustments for maltreatment reexposure and a less stringent covariate selection criterion. Effect size estimates were nearly identical for the model ignoring reexposure and the model addressing reexposure, consistent with the interpretation that the results did not differ meaningfully depending on whether children exposed to maltreatment prior to Age 4 and again after Age 4 were included or excluded from the study analyses. This indicates that early‐life CM has a long‐term impact on psychopathology symptoms. However, results from the pairwise contrasts showed the contamination group was also significantly different from the revised (i.e., no maltreatment) comparison group with regard to adolescent psychopathology symptoms, suggesting that maltreatment at any time is also related to more severe psychopathology symptoms in adolescence. Additionally, the robustness assessment for maltreatment reexposure resulted in negligible changes relative to the model that corrected contamination but not reexposure. This suggests that correcting contamination may have a stronger impact on the estimation of the effects of early‐life maltreatment on psychopathology relative to the effect of maltreatment reexposure.

The study results also did not differ meaningfully when an expanded set of covariates was included that may have occurred before the onset of maltreatment or been impacted by maltreatment. Indeed, there were similar patterns of findings regarding statistical significance and effect magnitude before and after adding additional covariates. Results from this robustness assessment suggest that, with a large enough sample and sufficient group sizes, it may be possible to exert less stringent criteria over covariate selection in propensity score methods without influencing statistical estimates. However, further research is needed to confirm this possibility and, in the meantime, existing guidance from the causal inference literature on the selection of confounders is recommended (Kainz et al., 2017).

There are several strengths of this study that advance the literature. First, the prospective, longitudinal nature of the research design allowed for an examination of the long‐term impact of early‐life maltreatment on adolescent psychopathology. Reliably demonstrating the statistical significance and magnitude of the effects of early‐life maltreatment may be particularly helpful in allocating intervention resources toward this developmental period, when the prevention of future psychopathology symptoms is likely to be most impactful. Second, this study focused on the impact of early life maltreatment on psychopathology symptoms reported via a transdiagnostic measure of psychopathology. CM increases the risk for multiple forms of psychopathology (Jaffe, 2017) that are highly comorbid (Teicher & Samson, 2013). The findings from this study further clarify the impact of early‐life maltreatment on a range of psychopathology symptoms, even after accounting for reexposure and the quality of covariates. Finally, this study extends existing research on contamination in CM studies (Scott et al., 2010; Shenk et al., 2022) by focusing on maltreatment exposure in this early developmental period.

Although the present study advances the study of contamination in the field of early life maltreatment, several limitations must be considered. First, this study examined two important dimensions of CM exposure—early‐life maltreatment and reexposure—when estimating the effects of maltreatment on adolescent psychopathology. However, we did not consider other CM dimensions, such as severity and subtype, that may differentially impact psychopathology following maltreatment (English, Upadhyaya, et al., 2005; Russotti et al., 2021). Researchers are encouraged to explore the potential role of other CM dimensions when examining the impact of contamination on psychopathology. Second, the present study classified self‐reported neglect as any endorsement of the most extreme rating on a dimensional measure of neglect. This method was chosen to create a binary indicator for self‐reported neglect that mirrored the binary indicators generated from self‐reported physical, sexual, and psychological abuse. Converting a dimensional measure of neglect into a binary indicator may have over‐ or underinflated the prevalence of neglect. However, other studies have used the same approach (Springer et al., 2007), which can approximate the prevalence of neglect in independent, national cohorts (see Shenk et al., 2022). Third, LONGSCAN data were collected between 1991 and 2012. It is possible that the prevalence and impact of contamination have shifted over the last few decades, and future work should examine contamination using more recent data. Finally, the generalizability of the results from this study is limited to the methods of the current study and characteristics of the LONGSCAN sample. For example, the current study used MMCS ratings of confirmed maltreatment to establish maltreatment and comparison groups, as well as self‐reports to identify contamination within the comparison group, which may generate different results when compared to studies using other methods. The results from this study, therefore, are best characterized as the effects of confirmed early‐life maltreatment on adolescent psychopathology before and after correcting contamination. Ultimately, the major takeaway from this study is that addressing contamination using multiple methods, including official case records and self‐report measures, can have important implications for the statistical significance and magnitude of the effects of early‐life CM.

The findings from the present study demonstrate that early‐life maltreatment was only related to higher levels of psychopathology in adolescence when contamination was corrected, this effect increased in magnitude after correcting contamination, and accounting for reexposure to maltreatment and the quality of covariates did not meaningfully alter these results. Study findings expand current literature on the longitudinal impact of maltreatment on psychopathology by offering a potential explanation for the replication failures and variation in effect magnitude seen across studies linking CM to psychopathology. Further, and importantly, these findings highlight that studies risk failing to detect or misclassifying the strength of maltreatment effects on psychopathology, among other outcomes, when they leave contamination unaddressed.

There are several implications of the study findings. First, future CM research would benefit from the use of a multimethod measurement strategy, such as official case records in combination with self‐ or caregiver reports, that is administered repeatedly throughout a study to detect contamination. Second, the first 4 years after birth may be a particularly important time to both prevent the onset and mitigate the impact of maltreatment on later psychopathology. By better estimating the effect of CM on psychopathology and identifying the developmental period in which the provision of prevention and treatment services may be the most impactful, researchers can leverage findings to provide more specific recommendations to policymakers with the goal of reducing the overall public health impact of CM. Third, the change in effect magnitude that resulted from correcting contamination, and the robustness of this change to maltreatment reexposure and the quality of covariates used to adjust the effects of maltreatment, can be used to inform CM researchers conducting statistical power analyses for future research, as well as policymakers on the severity of the effects of early life maltreatment on adolescent psychopathology. Finally, it will be important for future researchers to replicate a similar effect size magnitude for early life maltreatment on adolescent psychopathology when contamination is corrected. Such replication will lend stronger evidence of the effect of early life maltreatment on adolescent psychopathology.

## AUTHOR NOTE

The authors disclose receipt of the following financial support for the research, authorship, and/or publication of this article: The work was supported by the National Science Foundation (BCS‐2041333), the National Institutes of Health (R03HD104739), the National Institutes of Child Health and Human Development (T32HD101390), the Center for Healthy Aging at the Pennsylvania State University, and the Edna P. Bennett Faculty Fellowship in Prevention Research from The Pennsylvania State University.

The data used in this publication were made available by the National Data Archive on Child Abuse and Neglect, Cornell University, Ithaca, NY, and have been used with permission. Data from Longitudinal Studies of Child Abuse and Neglect (LONGSCAN) Assessments 0–12 were originally collected by Desmond K. Runyan, Howard Dubowitz, Diana J. English, Jonathan Kotch, Alan Litrownik, Richard Thompson, and Terri Lewis and the LONGSCAN Investigator Group. Funding for the project was provided by the Office on Child Abuse and Neglect (OCAN), Children's Bureau, Administration for Children and Families, Department of Health and Human Services. The National Center on Child Abuse and Neglect [NCCAN], under the Office of Human Services, funded this consortium of studies during the early years of data collection from April 1, 1991, until NCCAN became part of OCAN in 1998 (90CA1467, 90CA1481, 90CA1466, 90CA1458, 90CA1572, 90CA1569, 90CA1568, 90CA1566, 90CA1678,

90CA1681, 90CA1680, 90CA1676, 90CA1677, 90CA1679, 90CA1744, 90CA1745, 90CA1746, 90CA1747, 90CA1748, 90CA1749). The collector of the original data, the funder, the National Data Archive on Child Abuse and Neglect (NDACAN), Cornell University, and their agents and employees bear no responsibility for the analyses or interpretations presented here.

## OPEN PRACTICES STATEMENT

This study was preregistered at the Open Science Framework (OSF). The authors confirm that the study was registered prior to conducting the research and that the preregistration adheres to the disclosure requirements of the institutional registry. The preregistration can be found by following this link: https://doi.org/10.17605/OSF.IO/WGQJD. The data (LONGSCAN), data dictionaries, measure manuals, and a user's guide are available through protected access at the National Data Archive for Child Abuse and Neglect at Cornell University (https://www.ndacan.acf.hhs.gov/datasets/dataset‐details.cfm?ID=170; https://doi.org/10.34681/CXPJ‐5M96). To receive these materials, individuals can submit their contact information and a terms of use agreement (https://www.ndacan.acf.hhs.gov/datasets/request‐dataset.cfm). The statistical code for this project will be found on the OSF website.

## AUTHOR CONTRIBUTIONS

**Emily D. Dunning**: conceptualization, data curation, methodology, formal analysis, project administration, software, visualization, writing—original draft. **Chad E. Shenk**: conceptualization, supervision, writing—review and editing. **Zachary Fisher**: methodology, software. **Steffany J. Fredman**: Writing—review and editing. **John Felt**: software, validation.
